# Technique of retinal gene therapy: delivery of viral vector into the subretinal space

**DOI:** 10.1038/eye.2017.158

**Published:** 2017-08-18

**Authors:** K Xue, M Groppe, A P Salvetti, R E MacLaren

**Affiliations:** 1 Nuffield Laboratory of Ophthalmology, University of Oxford & Oxford Eye Hospital, Oxford University Hospitals NHS Foundation Trust, Oxford, UK

## Abstract

**Purpose:**

Safe and reproducible delivery of gene therapy vector into the subretinal space is essential for successful targeting of the retinal pigment epithelium (RPE) and photoreceptors. The success of surgery is critical for the clinical efficacy of retinal gene therapy. Iatrogenic detachment of the degenerate (often adherent) retina in patients with hereditary retinal degenerations and small volume (eg, 0.1 ml) subretinal injections pose new surgical challenges.

**Methods:**

Our subretinal gene therapy technique involved pre-operative planning with optical coherence tomography (OCT) and autofluorescence (AF) imaging, 23 G pars plana vitrectomy, internal limiting membrane staining with Membrane Blue Dual (DORC BV, Zuidland, Netherlands), a two-step subretinal injection using a 41 G Teflon tipped cannula (DORC) first with normal saline to create a parafoveal bleb followed by slow infusion of viral vector via the same self-sealing retinotomy. Surgical precision was further enhanced by intraoperative OCT (Zeiss Rescan 7000, Carl Zeiss Meditec AG, Jena, Germany). Foveal functional and structural recovery was evaluated using best-corrected Early Treatment Diabetic Retinopathy Study (ETDRS) visual acuity, microperimetry and OCT.

**Results:**

Two patients with choroideremia aged 29 (P1) and 27 (P2) years, who had normal and symmetrical levels of best-corrected visual acuity (BCVA) in both eyes, underwent unilateral gene therapy with the fellow eye acting as internal control. The surgeries were uncomplicated in both cases with successful detachment of the macula by subretinal vector injection. Both treated eyes showed recovery of BCVA (P1: 76–77 letters; P2: 84–88 letters) and mean threshold sensitivity of the central macula (P1: 10.7–10.7 dB; P2: 14.2–14.1 dB) to baseline within a month. This was accompanied by normalisation of central retinal thickness on OCT.

**Conclusions:**

Herein we describe a reliable technique for subretinal gene therapy, which is currently used in clinical trials to treat choroideremia using an adeno-associated viral (AAV) vector encoding the *CHM* gene. Strategies to minimise potential complications, such as avoidance of excessive retinal stretch, air bubbles within the injection system, reflux of viral vector and post-operative vitritis are discussed.

## Introduction

Retinal gene therapy offers our best hope for treating inherited retinal dystrophies in the foreseeable future based on successful proof-of-principle in animal models and promising data from human clinical trials. Adeno-associated viral (AAV) vector based gene therapy for *RPE65*-associated Leber congenital amaurosis (LCA) and choroideremia (CHM) have entered Phase III and II clinical trials respectively. In both cases, demonstrating long-term improvements in visual function in those patients in whom early treatment effects were observed.^1, 2, 3, 4^ AAV gene therapies for other monogenic retinal disorders are under investigation, eg MERTK-associated retinitis pigmentosa,^5^ X-linked retinoschisis (XLRS), *CNGA3*-associated achromatopsia and *ND4*-associated Leber hereditary optic neuropathy (LHON).^6^ In addition, gene therapy has been used to create sustained ectopic production of a secreted anti-vascular endothelial growth factor protein as an alternative way of treating wet age-related macular degeneration.^7^

Unlike conventional drug treatments, retinal gene therapy is a complex biological treatment whose efficacy could depend on a multitude of factors, including optimised AAV vector design, high quality vector production and intervention at an appropriate stage of the disease.^8^ The least unpredictable of all the variables is the surgical delivery to the subretinal space, which must minimise trauma but maximise viral transduction of the target cells. Reflux of the vector suspension into the vitreous cavity will, by definition, reduce the subretinal dose applied to the target cells. Furthermore, intravitreal AAV particles may stimulate an immune response to the viral capsids (vitritis), which may further degrade the number of effective AAV particles in the subretinal space. Although intravitreal injection of vector can be used to treat diseases primarily affecting the inner retina (eg, Müller cells in XLRS and ganglion cells in LHON), it is disadvantaged by vector dilution within the vitreous cavity. A modification of this approach by injecting under the internal limiting membrane (ILM) may avoid some of these problems.^9^ Here we focus on the technique of subretinal gene therapy, which offers a targeted approach to treating outer retinal degenerations caused by genetic mutations in the retinal pigment epithelium (RPE) and photoreceptors. Administration of AAV vector into the potential space between the photoreceptor outer segments and the RPE allows treatment of a specific area of the retina (eg, the macula), generates a high local concentration of vector to enhance the proportion of cells transduced, and helps to evade adaptive immune responses. Furthermore, the hydrostatic force of the subretinal injection will pump vector suspension into the extracellular space of the outer retina, thereby providing a potential reservoir of AAV particles for further transduction after the initial subretinal fluid has been reabsorbed.

## Materials and methods

### Pre-operative assessment

As part of surgical planning prior to undergoing gene therapy, choroideremia patients with confirmed genetic mutations in the *CHM* gene were assessed with a range of baseline visual function tests as well as retinal imaging. These included best-corrected visual acuity (BCVA) using the ETDRS chart at 4 m, MAIA microperimetry (Centervue, Padova, Italy) using the 10-2 pattern and 4-2 dB bracketing strategy under mesopic condition (background luminance 1.3 cd/m^2^), spectral domain OCT (Spectralis, Heidelberg Engineering, Heidelberg, Germany) and fundus autofluorescence (AF, BluePeak, Spectralis).^10^

### AAV vector

The AAV vector for choroideremia gene therapy consists of a chicken *β*-actin (CBA) promoter, human *CHM* cDNA encoding Rab-escort protein 1 (REP1), bovine poly(A) signal and a modified Woodchuck hepatitis virus post-transcriptional regulatory element (WPRE), packaged in the AAV serotype 2 capsid, as described previously.^3, 10^ The subretinal dose of AAV.REP1 vector was 0.1 ml of a 10^12^ genome particles (gp) per ml solution containing 0.001% PF-68, a surfactant shown to reduce adhesion of AAV viral capsids to the materials of the injection system.^11^

### Subretinal injection

The technique of subretinal delivery of AAV vector described below is currently used in two interventional clinical trials of gene therapy for choroideremia: (1) An open label dose escalation Phase 1 clinical trial of retinal gene therapy for choroideremia using an adeno-associated viral vector encoding Rab-escort protein 1 (ClinicalTrials.gov identifier: NCT01461213), and (2) An open label Phase 2 clinical trial of retinal gene therapy for choroideremia using an adeno-associated viral vector encoding REP1 (ClinicalTrials.gov identifier: NCT02407678).

As part of the trial protocol, peri-operative corticosteroid (oral prednisolone) was given, starting two days prior to surgery at 1 mg/kg for 10 days, then 0.5 mg/kg for 7 days, 0.25 mg/kg for 2 days and then 0.125 mg/kg for 2 days. For an 80 kg man, the doses per day would be equivalent to 80, 40, 20 and 10 mg. Gastric protection was given in the form of oral omeprazole 20 mg twice daily for the duration of the corticosteroid regime.

The surgery consisted of a standard three-port 23-gauge pars plana vitrectomy, induction of posterior vitreous detachment (if not already present), core and peripheral vitrectomy, and ILM staining with Membrane Blue Dual (DORC, Zuidland, The Netherlands). Macula detachment was induced by subretinal injection of a balanced salt solution (BSS). The injection device was an extendible 41 G polytetrafluoroethylene (Teflon) blunt-tipped subretinal cannula (DORC) connected to the viscous fluid control (VFC) port of the Alcon Constellation Vision System (Alcon, Fort Worth, TX, USA). The infusion pressure was controlled via a foot-pedal with the maximum limit set to the minimum that would produce a continuous flow of fluid rather than a stream of droplets—generally this was 12–16 psi.

After flushing and testing for bubbles by immersing the tip of the cannula into a BSS dish outside of the eye, the needle was retracted and introduced through one of the ports. The needle tip was then advanced and guided to the retina in the region of the superior vascular arcades, but avoiding the blood vessels, where the subretinal injection was initiated. Once propagation of the subretinal BSS bleb towards the fovea was seen and confirmed by intraoperative OCT (Zeiss Rescan 7000, Carl Zeiss Meditec AG, Jena, Germany) displayed within the surgeon’s microscope eyepiece as overlay, the BSS needle was retracted.

Next 0.3 ml of AAV.REP1 vector was loaded into a 1 ml syringe (Beckton Dickinson, Oxford, UK), fitted with a disposable dual-bore 41 G/23 G Teflon tipped cannula (DORC) and connected to the Constellation system via a custom syringe lock and standard VFC tubing (Figure 1). Care was taken during BSS and vector loading to purge all air bubbles from the injection systems as described previously.^11^ As with the initial BSS injection, the primed vector injection system was first tested in a pot of saline under the microscope prior to entering the eye. A maximum dose of 0.1 ml of the AAV vector was slowly infused into the subretinal bleb through the existing retinotomy under foot-pedal control. This slow approach avoided over-stretch of the retina because there is often insufficient subretinal volume for 100 *μ*l of fluid in the end-stage CHM patients. Occasionally an additional retinotomy was made with the 41 G cannula to fully detach a large ‘retinal island’ or treat a non-contiguous retinal island as seen on AF imaging. The progression of retinal detachment, including detachment of the fovea, was carefully monitored by intraoperative OCT. In some cases where there were areas of focal thinning, heavy liquid was used to provide internal tamponade of the retina, similar to the technique described by Maguire to protect the thin fovea in LCA patients.^8^ After delivery of vector into the subretinal space, the vitreous cavity was irrigated (to clear any refluxed free floating AAV particles). In most cases the eye was left fluid filled (to minimise vector reflux and risk of cataract formation) and all sclerostomies were sutured with 8-0 polyglactin (Vicryl, Ethicon, Johnson and Johnson, New Brunswick, NJ, USA).

## Results

The typical surgical technique for subretinal gene therapy and perioperative findings are illustrated using data and images from two recent CHM patients (both brothers) aged 29 (Patient 1) and 27 (Patient 2) years. Both underwent unilateral gene therapy on the same day as part of a Phase II clinical trial, with the fellow eye acting as an internal control. The patients had normal and symmetrical levels of BCVA in both eyes at baseline—76 letters OD (treated) *versus* 79 OS (control) in P1, and 87 OD (control) *versus* 84 OS (treated) in P2 (Supplementary Table I)—but with significant loss of peripheral visual fields as demonstrated by microperimetry. The surgeries were uncomplicated in both cases with successful detachment of the residual central retinal ‘island’ by subretinal vector injection. Intraoperative OCT enabled early detection of the build-up of subretinal fluid at the injection site during bleb initiation (while excluding suprachoroidal injection), and provided early indication of the direction of propagation of the bleb, thus allowing the surgeon to adjust the position of the cannula as required. It also helped to confirm foveal detachment in both cases, indicating that the retinal structure responsible for visual acuity was targeted successfully (Figure 2).

The preoperative baseline assessments used for surgical planning were BCVA, MAIA microperimetry, OCT and AF imaging. These helped to determine the functional and structural integrity of the fovea, and the area of preserved photoreceptors (ellipsoid zone on OCT) and RPE cells (AF ‘islands’) to which gene therapy could be applied, since the AAV vector is designed to replace the non-functioning *CHM* gene within surviving cells but would not be expected to rescue areas where the cells have already been lost.^12^ OCT was also used to rule out cystoid macular oedema, retinoschisis or choroidal neovascularisation at baseline, which could occasionally associate with CHM. The 10-2 MAIA microperimetry assessed the threshold sensitivities of the central 20° field (covered by 68 points) with ‘Stable’ fixation stability achieved in all cases (Figure 3). The areas with detectable threshold sensitivities (>2.0 dB, coloured dots) plotted over the inbuilt confocal scanning laser ophthalmoscope (cSLO) image could be seen to correspond to the area of residual autofluorescence, whereas areas with non-detectable sensitivities (<2.0 dB, black dots) corresponded to atrophic regions lacking autofluorescence. The fixation points (shown by cluster of fine green dots on the microperimetry maps) corresponded to the areas with the greatest threshold sensitivities (up to 16.0 dB) and the location of the foveal dip on the OCT.

Post-operatively, the retinotomies self-sealed without sequelae and subretinal fluid resolved within 24 hours (Figure 4). Structural recovery of the retina following iatrogenic detachment of the macula generally occurred within one month (Figure 4 and Supplementary Table S1). In both patients, the mean central retinal thickness (of the 1 mm^2^ ETDRS circle) and retinal volume (from the ILM to the Bruch’s membrane within the 1 mm^2^ ETDRS circle) were increased from baseline on day 1, partially resolved by 1 week and returned to baseline by 1 month post-operatively. Since the retinal oedema was induced by the positive pressure subretinal injection (as seen with the intraoperative OCT imaging), its persistence on post-operative day 1 indicates that intra-retinal fluid persists for longer than subretinal fluid. This may have additional advantages for retinal transduction, particularly for photoreceptors, as vector particles will be surrounding and in direct contact with outer nuclear layer cells long after the subretinal fluid has been reabsorbed by the RPE. This is a potential advantage in using the subretinal compared with intravitreal approach.^13^ The resolution of retinal oedema by 1 month following gene therapy was associated with preservation of the ‘retinal islands’ on AF imaging (Figure 3). Functional recovery was demonstrated by return of BCVA to baseline levels in both patients by 1 month follow-up—77 letters OD (treated) *versus* 83 OS (control) in P1, and 87 OD (control) *versus* 88 OS (treated) in P2. In addition, the mean central retinal sensitivities of the treated eyes on microperimetry also returned to baseline at 1 month.

## Discussion

While many early gene therapy clinical trials have recruited patients with advanced disease, the reassuring safety and efficacy data emerging from these trials would suggest that early intervention might be more beneficial to preserve retinal cells prior to a potentially irreversible stage of structural disorganisation. In choroideremia, we have previously shown that the edge of photoreceptor loss as seen on OCT closely follows the extent of RPE loss on AF image, suggestive of mutually dependent cell survival, although some photoreceptors appeared to persist beyond the edge of RPE loss in the form of outer retinal tubulations.^14^ Moreover, our natural history study showed the mean lifetime rate of shrinkage of the AF area in CHM to be 7.7% per year, meaning that the absolute area of retina lost per year is greater at younger age.^15^ Prior to subretinal gene therapy, combined OCT and AF imaging are essential for confirming retinal structural integrity within the treatment zone to tolerate a safe subretinal approach. It is important to identify areas of excessive retinal thinning, which could open up under subretinal pressure to form a secondary retinal hole, leading to failure of BSS/vector bleb propagation, egress of vector and potentially reduced visual acuity. A bubble of heavy liquid may be placed over an area of excessive retinal thinning during the subretinal injection to help prevent the formation of a secondary retinal hole. Microperimetry and OCT are also particularly informative for identifying eyes in which the foveal photoreceptors lie immediately on the advancing ‘frontier’ of RPE loss since these might be particularly susceptible to visual acuity fluctuations following an iatrogenic retinal detachment. It remains unclear whether the cells around the edge of the residual AF islands are amenable to rescue by gene supplementation. Continued degeneration of these ‘border cells’ could potentially lead to a paradoxical drop in vision in the short-term but slowing of disease progression in the long-term.

The surgical technique of subretinal gene therapy builds upon established subretinal procedures such as subretinal tissue plasminogen activator (tPA) injection and macular translocation surgery. However, specific considerations must be made to adapt the technique to the retinal characteristics in hereditary retinal dystrophies. Unlike subretinal tPA injection where the subretinal space has already been opened up and demarcated by blood, inducing a clean foveal detachment in choroideremia is unpredictable due to the subretinal adhesions surrounding the AF islands which strongly resist extension of the iatrogenic detachment. Also unlike subretinal tPA patients who have underlying macular pathology, choroideremia patients undergoing gene therapy often have normal BCVA (6/6), which raises new challenges to minimise foveal stretch during vector delivery. Depending on the approximate diameter of the AF island in each eye, the maximum volume (*V*_*max*_) of the subretinal bleb could be approximated using the spherical cap formula (Figure 5)^16^- an injection volume beyond *V*_*max*_ would be expected to cause either retinal stretch (with resulting mechanical disruption of nerve fibres and neuronal synapses) and/or reflux from the retinotomy Knowing *V*_*max*_ would allow the surgeon to predict the maximum dose for a safe subretinal injection. It would also inform clinical trial design since it may not be practical to define a standard dose volume for all choroideremia patients, but rather tailor the dose to the AF area being treated.

The incorporation of foot-pedal control and intraoperative OCT helps to minimise excessive retinal stretch by allowing the surgeon to monitor in real-time the amount of the retinal elevation induced by the BSS and vector injections while titrating the infusion pressure by foot. Since the retinotomy may become enlarged by the cannula tip during the two-step injection process, visualisation of the rise and fall of the bleb on the intraoperative OCT during vector injection helped to indicate that the vector was filling the subretinal space rather than refluxing directly into the vitreous cavity. The sensitivity of OCT also meant foveal detachment could be achieved with shallow subretinal fluid only (rather than gross foveal elevation visible under the microscope), which minimised foveal stretch. Furthermore, an intraoperative OCT volume scan taken at the end of vector injection would enable the calculation of final bleb volume, giving an indication of the dose given. This has implications for the efficacy of gene therapy, and would be an important consideration when evaluating the outcomes of clinical trials. Since tissue stress would be expected to be highest adjacent to the retinotomy during the induction of the detachment, the initial retinotomy should ideally be placed away from the fovea or areas of retinal thinning, as described previously.^10^ In our experience, a retinotomy located close to the superior vascular arcade at one of the tips of the AF island generally works well in allowing propagation of the detachment towards the fovea. Although 41 G retinotomies are ultimately self-sealing, it may be temporarily leaky especially if enlarged during the injection process, enabling some subretinal fluid to leak out during the recovery period. Since we do not use any air or gas tamponade at the end of the procedure, a superiorly placed retinotomy might allow less vector leakage than an inferior one if the patient postured upright since the vector suspension is denser than BSS.

Another potential source of undesirable retinal stretch during subretinal injection is the presence of air bubbles within the needle or syringes. Sudden forceful expansion of a large air bubble as it emerges from the pressurised injection system into a restricted subretinal bleb could lead to acute retinal stretch and possible macular hole formation, especially in the thin degenerate retina of patients with retinal dystrophies.^17, 18^ Stringent efforts were made to purge the BSS and vector syringes of any visible air bubble prior to injection. However, microscopic air bubbles do occasionally appear which were thought have been trapped by the internal ridges at the interphase between the needle shaft and plastic mount.^11^ These microscopic bubbles appeared to be harmless and had a tendency to sit directly under the retinotomy, which was helpful for reducing potential vector reflux during the injection process.

The risk of AAV-induced post-operative vitritis was reduced in a several ways. Perioperative systemic corticosteroid was used to induce transient immunosuppression, which has been well tolerated by all patients treated in several retinal gene therapy trials to date.^2, 10, 17^ The dual-bore design of the subretinal cannula allows egress of vitreous fluid from near the cannula tip to the outside due to the increased intraocular pressure during subretinal injection. This creates a passive suction effect akin to the flute cannula, which may help to remove viral particles refluxing from the retinotomy in the vicinity. At the end of the procedure, the vitreous cavity was irrigated gently to remove any refluxed vector particles.

Irrespective of the therapeutic effects of the gene therapy in the long-term, the retinal structural and functional recovery to baseline seen at one month would suggest that iatrogenic macular detachment can be safely performed as part of the subretinal approach to gene therapy. Future development to make subretinal gene therapy even safer might involve prolonged mechanical infusion of BSS and vector, or a precise one-step OCT-guided subretinal infusion of vector. A robotically assisted retinal surgery system to enable ‘hands-free’ slow subretinal infusion may also achieve these goals. Ultimately developments in gene therapy surgery must go hand-in-hand with improved vector design. It is agreed that a 90% intravitreal reflux of vector suspension during non-optimal subretinal injection can be compensated by using a vector that is ten times more efficacious. But improving the surgical technique to increase vector delivery from 10 up to 100% is far easier and more logical.


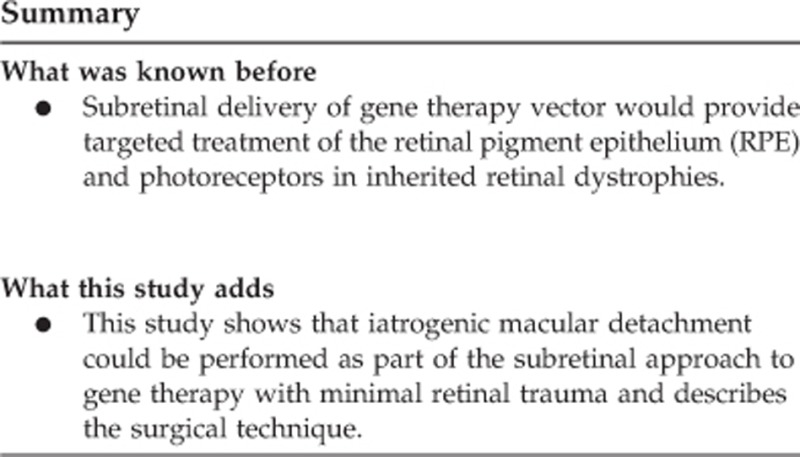


## Figures and Tables

**Figure 1:**
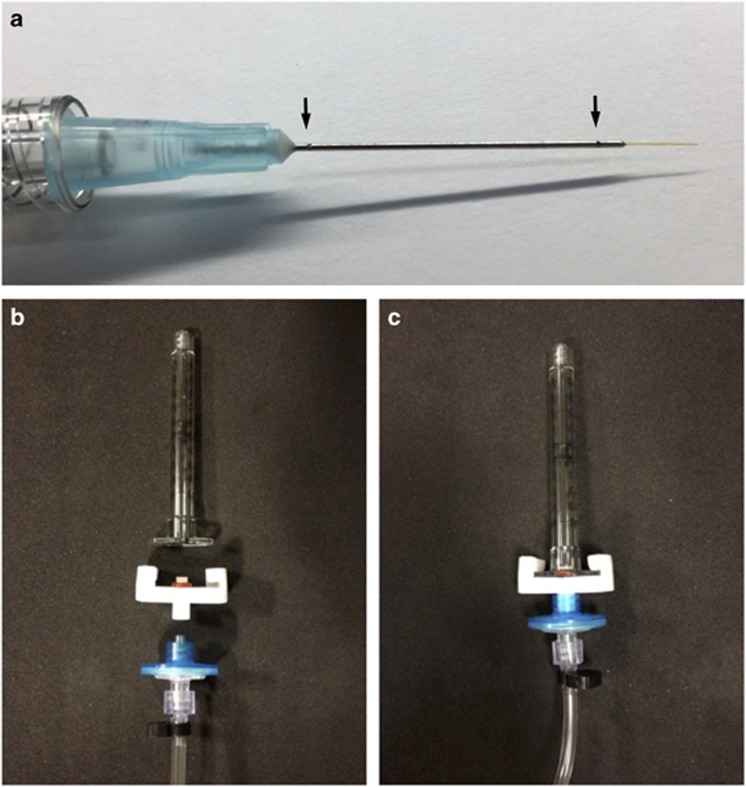
Subretinal gene therapy injection system. (a) A dual-bore 41 G blunt polytetrafluoroethylene (Teflon) tipped cannula mounted within a 23 G steel shaft (DORC, Zuidland, The Netherlands). Arrows indicate proximal and distal pressure relief holes along the shaft, which allow evacuation of excess vitreous fluid out of the eye during the injection. (b) Components of the subretinal injection system consisting of a 1 ml BD syringe, custom syringe lock with a rubber O-ring, air filter and polyethylene connection tubing (Alchimia, Ponte S Nicolò, Italy). (c) Assembled injection system.

**Figure 2:**
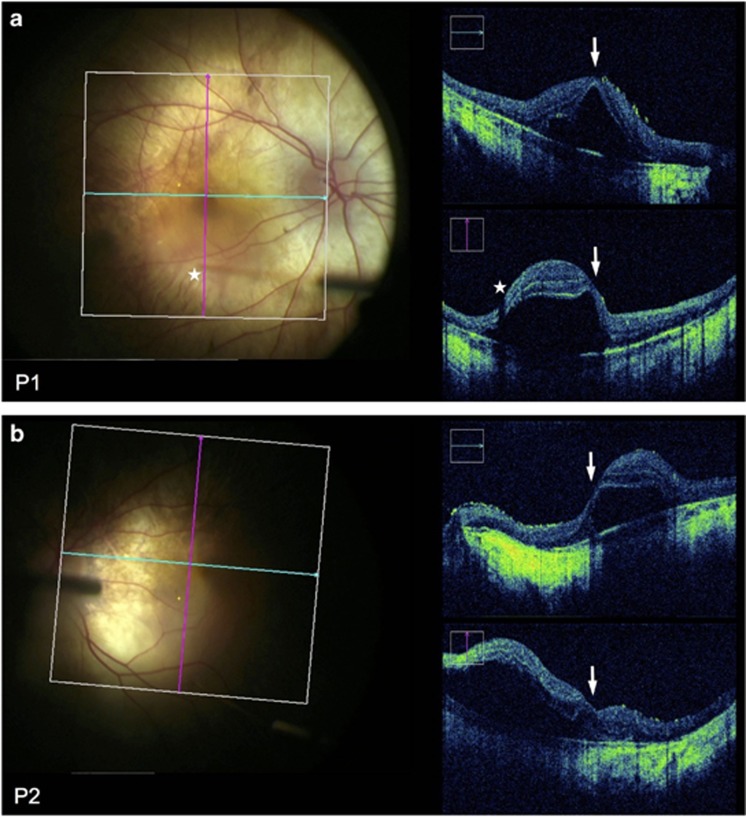
OCT-guided subretinal injection. Microscope photograph and simultaneous intraoperative OCT (Zeiss Rescan 7000, Carl Zeiss Meditec AG, Jena, Germany) during subretinal gene therapy for choroideremia in Patient 1 OD (a) and Patient 2 OS (b). Blue and pink grid lines indicate the locations of horizontal and vertical OCT B-scans respectively. The fovea could be seen to be detached in both cases (arrows). Stars indicate the tip of the subretinal cannula (seen out of focus) and the corresponding shadow cast on the OCT.

**Figure 3:**
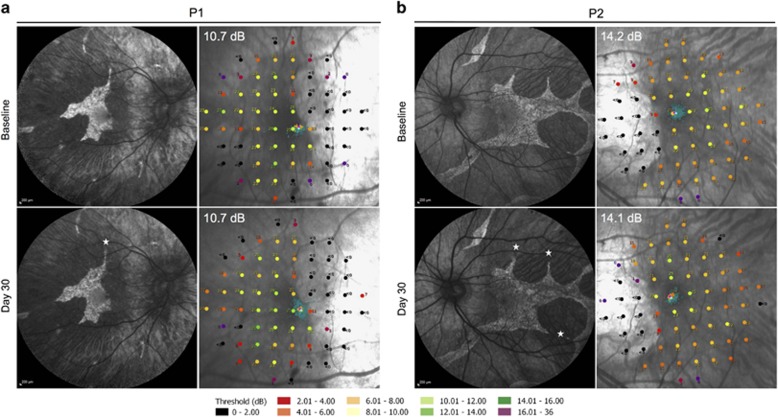
Preservation of autofluorescence (AF) island and central retinal sensitivity following subretinal gene therapy. Baseline and 30 days post-operative AF images and 10-2 MAIA microperimetry of the treated eye of Patient 1 (a) and Patient 2 (b) are shown. Stars indicate the locations of retinotomies (one in P1 and three in P2) performed during the subretinal BSS and vector injections, which successfully detached the central retinal island in each case. The threshold retinal sensitivities of the central 20° of the macula were represented by 68 colour-coded points superimposed on the cSLO image. The points of fixation are shown as clusters of fine green dots.

**Figure 4:**
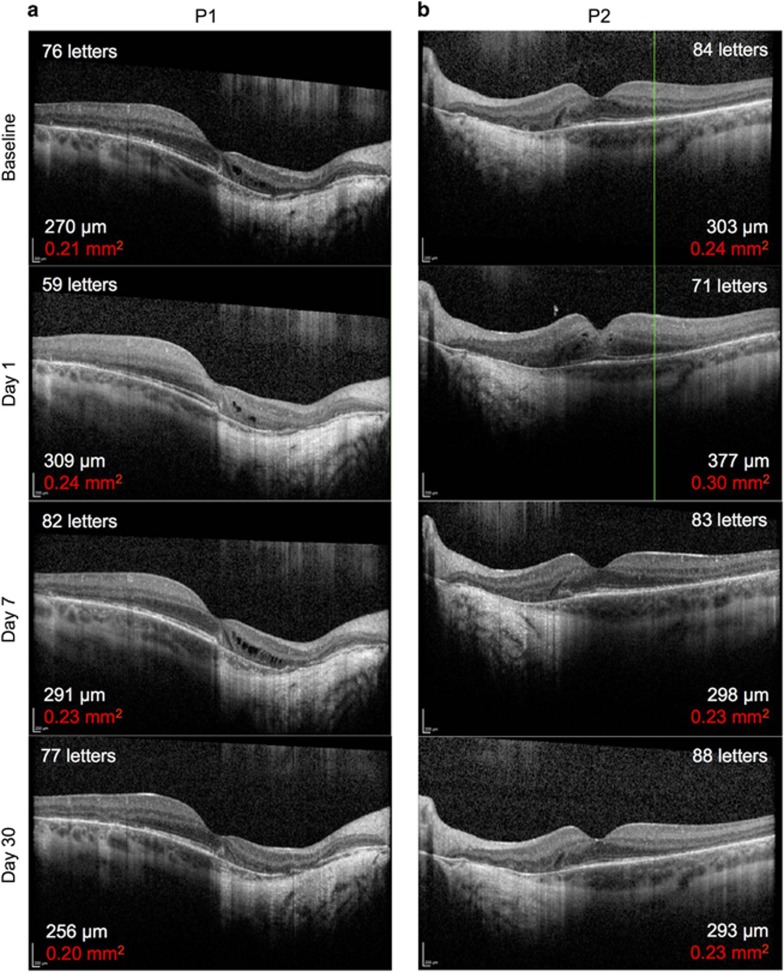
Resolution of subretinal fluid and recovery of visual acuity following subretinal gene therapy. Baseline and post-operative day 1, 7 and 30 foveal OCT images from Patient 1 (a) and Patient 2 (b) as described in Figures 2 and 3. The best-correct visual acuity (number of ETDRS letters) at each time-point is shown in the top corner of each OCT image. The mean central retinal thickness of the 1 mm^2^ ETDRS circle (in *μ*m) and retinal volume from the ILM to the Bruch’s membrane within the 1 mm^2^ ETDRS circle (in mm^2^, red) are shown in the bottom corner of each OCT image. No significant changes in mean central retinal thickness or volume were observed in the fellow eye of either patient (Supplementary Table I).

**Figure 5:**
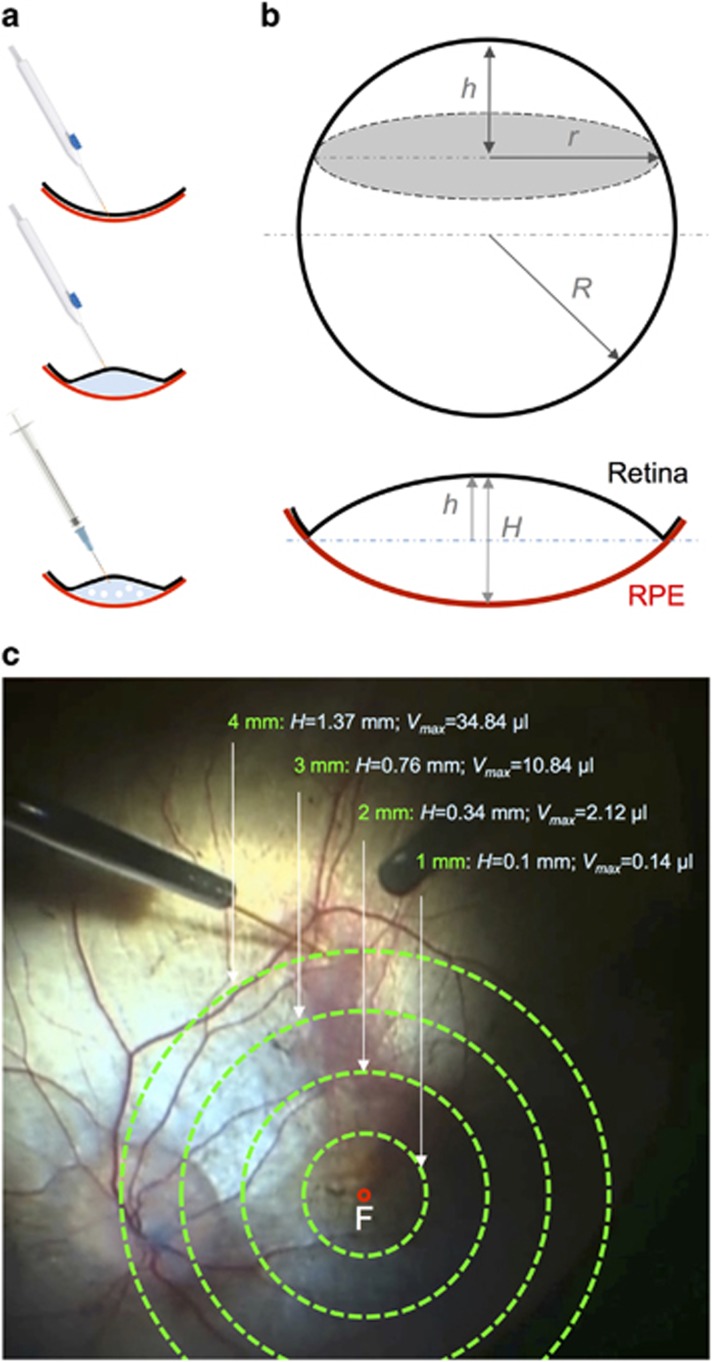
The principle of subretinal bleb formation during gene therapy for choroideremia. (a) A schematic drawing of key surgical steps consisting of the initiation of retinal detachment by subretinal injection of basic saline solution (BSS) using an extendible 41 G cannula (DORC), a necessary step to overcome the abnormally adherent retina of patients with outer retinal degenerations. The AAV vector solution is then loaded into a 1 ml syringe and slowly infused into the subretinal bleb through the same retinotomy using a dual-bore 41 G cannula (DORC). (b) The spherical cap formula can be used to calculate the height of the subretinal bleb (*H*=2 *h*) in relation to the distance of the retinotomy from the fovea (*r*) and the radius of the whole eye (*R*, assumed to be half of the average axial length of 24 mm). (c) If the retina was assumed to ‘flip’ from concave to convex without undergoing any elastic stretch during a subretinal injection, for each diameter of subretinal bleb (*r*=1, 2, 3 or 4 mm), the maximum bleb volume (*V*_max_) was calculated to be 0.14, 2.12, 10.84 and 34.84 *μ*l, respectively.
