# Supplementary material for: Technique of retinal gene therapy: delivery of viral vector into the subretinal space
Source: Eye (Lond). 2017 Aug 18;31(9):1308–16. doi: 10.1038/eye.2017.158 (PMC5601444; doi:10.1038/eye.2017.158)
Supplement: Supplementary Table I [file eye2017158x1.docx]

**Supplementary Table I.** Foveal functional and structural recovery following subretinal gene therapy. Patients 1 and 2 correspond to the same individuals shown in Figures 2-4. Red denotes data from the eye treated with subretinal gene therapy (OD for P1 and OS for P2).

| **Patient 1** | | | | | | |
| --- | --- | --- | --- | --- | --- | --- |
|  | BCVA (OD), ETDRS letters | BCVA (OS), ETDRS letters | Central 1mm^2^ mean retinal thickness (OD), μm | Central 1mm^2^ mean retinal thickness (OS), μm | Central 1mm^2^ total retinal volume (OD), mm^3^ | Central 1mm^2^ total retinal volume (OS), mm^3^ |
| Baseline | 76 | 79 | 270 | 311 | 0.21 | 0.24 |
| Day 1 | 59 | 77 | 309 | 310 | 0.24 | 0.24 |
| Day 7 | 82 | 85 | 291 | 310 | 0.23 | 0.24 |
| Day 30 | 77 | 83 | 256 | 305 | 0.20 | 0.24 |
| **Patient 2** | | | | | | |
| Baseline | 87 | 84 | 306 | 303 | 0.24 | 0.24 |
| Day 1 | 77 | 71 | 306 | 377 | 0.24 | 0.30 |
| Day 7 | 83 | 83 | 303 | 298 | 0.24 | 0.23 |
| Day 30 | 87 | 88 | 304 | 293 | 0.24 | 0.23 |
